# Redox Regulation of Inflammatory Processes Is Enzymatically Controlled

**DOI:** 10.1155/2017/8459402

**Published:** 2017-10-08

**Authors:** Inken Lorenzen, Lisa Mullen, Sander Bekeschus, Eva-Maria Hanschmann

**Affiliations:** ^1^Department of Structural Biology, Institute of Zoology, Kiel University, Kiel, Germany; ^2^Brighton and Sussex Medical School, Falmer, Brighton, UK; ^3^Leibniz-Institute for Plasma Science and Technology (INP Greifswald), ZIK plasmatis, Greifswald, Germany; ^4^Department of Neurology, Medical Faculty, Heinrich-Heine University, Düsseldorf, Germany

## Abstract

Redox regulation depends on the enzymatically controlled production and decay of redox active molecules. NADPH oxidases, superoxide dismutases, nitric oxide synthases, and others produce the redox active molecules superoxide, hydrogen peroxide, nitric oxide, and hydrogen sulfide. These react with target proteins inducing spatiotemporal modifications of cysteine residues within different signaling cascades. Thioredoxin family proteins are key regulators of the redox state of proteins. They regulate the formation and removal of oxidative modifications by specific thiol reduction and oxidation. All of these redox enzymes affect inflammatory processes and the innate and adaptive immune response. Interestingly, this regulation involves different mechanisms in different biological compartments and specialized cell types. The localization and activity of distinct proteins including, for instance, the transcription factor NF*κ*B and the immune mediator HMGB1 are redox-regulated. The transmembrane protein ADAM17 releases proinflammatory mediators, such as TNF*α*, and is itself regulated by a thiol switch. Moreover, extracellular redox enzymes were shown to modulate the activity and migration behavior of various types of immune cells by acting as cytokines and/or chemokines. Within this review article, we will address the concept of redox signaling and the functions of both redox enzymes and redox active molecules in innate and adaptive immune responses.

## 1. Concept of Redox Signaling

**Figure 1:**
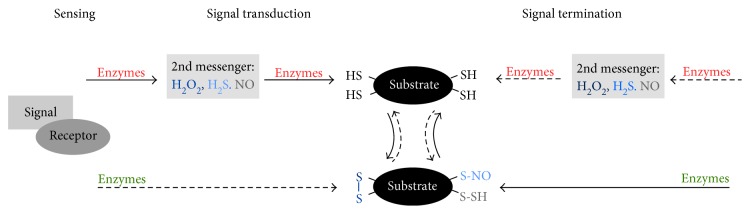
Concept of redox signaling. A signal is sensed by its receptor, inducing the enzymatic catalyzed production and release of second messengers (e.g., H_2_O_2_, NO, and H_2_S). These activate a cascade of transducing proteins via specific oxidative modifications at Cys residues (e.g., disulfide formation, nitrosylation, and sulfhydration). The effector molecule induces the biological response. A signal can also induce the reduction of distinct Cys residues. The activated signaling cascade becomes terminated, and cysteinyl modifications are reversed. The involved thiol groups are known as thiol switches. Their reduction (green), as well as their oxidation (red) are regulated by different enzymes.

Cells can receive and respond to distinct signals and environmental changes; they can send out signals in order to communicate with other cells. Signal transduction can depend on intracellular or membrane-bound receptors that have the ability to bind specific ligands that induce particular signaling cascades involving second messengers and rapid, reversible posttranslational modifications of transducer and effector proteins. Some signaling molecules can pass the plasma membrane and directly interact with specific targets. In the case of redox regulation, we can distinguish between different spatiotemporal modifications of cysteine residues, such as the formation of inter- or intramolecular disulfide bridges, S-glutathionylation by the formation of a mixed disulfide with glutathione (GSH), S-nitrosylation in the presence of nitric oxide (NO), the formation of sulfenic acid, for example, in the presence of hydrogen peroxide (H_2_O_2_), or the formation of S-sulfhydration by hydrogen sulfide (H_2_S). All these modifications modify the redox state of a particular thiol group and can affect a protein in terms of structure, localization, and/or activity [1] (Figure 1()). These regulatory thiol groups are known as thiol switches [2]. Interestingly, redox modifications also affect other posttranslational modifications, essential for signal transduction, for instance, phosphorylation. Redox signaling occurs upon specific stimuli and is localized in specific compartments or confined areas within a cellular compartment. The signal is sensed by a particular receptor, inducing the production and release of second messengers such as H_2_O_2_, NO, and H_2_S. Interestingly, not all reactive oxygen, nitrogen, and sulfur species are considered signaling molecules. This is due to their high reactivity towards a wide range of unspecific targets including various biomolecules, such as DNA, lipids, and proteins, and the lack of regulation of their production and decay. The hydroxyl radical, for instance, is nonenzymatically produced in the Fenton reaction and reacts with basically any molecule due to its high reactivity and lack of specificity [1]. Similarly, peroxynitrite is not considered a second messenger, because it is spontaneously formed by the reaction of nitric oxide with superoxide and a strong oxidizing agent with a second-order rate constant of 10^10^ M^−1^·s^−1^ that also oxidizes various biomolecules (reviewed in [3, 4]). H_2_O_2_, NO, and H_2_S activate effector molecules that induce a certain biological response via specific transducing molecules including redox couples, for example, GSH and oxidized glutathione (GSSG) and enzymes, for example, oxidoreductases of the thioredoxin (Trx) family. In the absence of the signal, the activated signaling cascade becomes terminated and cysteinyl modifications are reversed. These thiol switches have been predicted to play a role in almost every signaling cascade and are therefore essential for all biological processes. Obviously, physiological redox signaling is highly regulated and depends on the controlled oxidation as well as the specific reduction of substrates [1, 5]. The dysregulation or even disruption of redox signaling has been described as oxidative stress, a hallmark of various pathologies [6].

**Figure 2:**
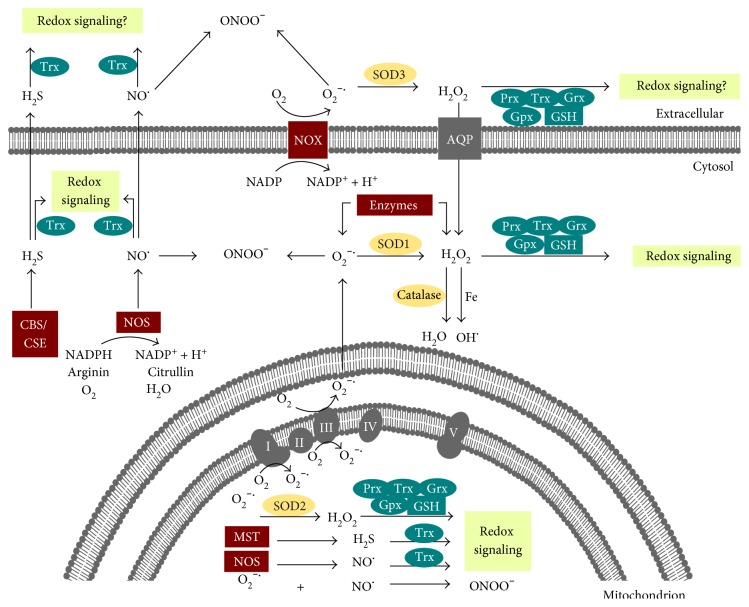
Redox regulation is enzymatically controlled. Illustration of cellular and extracellular enzymes that (i) generate redox active species (red), (ii) decompose reactive species, and are classified as antioxidants (yellow) or (iii) participate in redox signaling (blue). In the cytosol, superoxide (O_2_^−^) and hydrogen peroxide (H_2_O_2_) can be produced by specific enzymes; the cytosolic SOD1 can convert O_2_^−^ to H_2_O_2_. Moreover, the NADPH and oxygen-dependent membrane protein NADPH-oxidase (NOX) can produce O_2_^−^ that is converted to H_2_O_2_ by extracellular SOD3. The latter can cross the membrane via simple diffusion and aquaporins. H_2_O_2_ can participate in cell signaling as a second messenger via the action of the thioredoxin family members peroxiredoxin (Prx), thioredoxin (Trx), glutaredoxin (Grx), and glutathione peroxidases. These enzymes are NADPH- and mostly glutathione- (GSH-) dependent. H_2_O_2_ can also be reduced to water by the peroxidase catalase, which is mainly located in peroxisomes. However, in the presence of free iron, the highly reactive and damaging hydroxyl radical (OH^•^) is formed from H_2_O_2_ via the Fenton reaction. Nitric oxide (NO) is generated by cytosolic NO-synthase (NOS) and hydrogen sulfite (H_2_S) by the enzymes cystathionine *β*-synthase (CBS) and cystathionine *γ*-lyase (CSE). Both constitute second messengers that can participate in redox signaling via the action of Trx. Note that peroxynitrite (ONOO^−^) can spontaneously form in the presence of O_2_^−^ and NO, inducing irreversible modifications of various biomolecules and thus not participating in redox signaling. In mitochondria, complexes I and III of the mitochondrial respiratory chain produce superoxide (O_2_^−•^). Superoxide dismutase 2 (SOD2) converts O_2_^−^ to H_2_O_2_. Mitochondrial NOS and 3-mercaptopyruvate sulfurtransferase (MST) produce NO and H_2_S, respectively. Mitochondrial H_2_O_2_, NO, and H_2_S can participate in redox signaling. Similar to the cytosol, ONOO^−^ and OH^•^ can also be formed in the mitochondria. In the extracellular environment, NOX and SOD3 produce O_2_^−^ and H_2_O_2_ and the intracellularly produced NO and H_2_S can cross the plasma membrane. Members of the Trx family of proteins are found extracellular. Therefore, the intracellular concept of redox signaling might also occur in the microenvironment of the cell.

As mentioned above, the production and release of redox active molecules are regulated by enzymes that are located in various cellular compartments and also in the extracellular space (Figure 2()). Complexes I and III of the respiratory chain and enzymes such as nicotinamide adenine dinucleotide phosphate- (NADPH-) oxidases (NOX) and xanthine oxidase produce superoxide (O_2_^•−^). Superoxide dismutases (SOD) convert O_2_^•−^ into H_2_O_2_. Different peroxidases, including catalase and the Trx family members peroxiredoxins (Prxs) and glutathione peroxidases (Gpx), reduce H_2_O_2_ to water. NO is synthesized by one of the three isoforms of nitric oxide synthase (NOS), that is, neuronal nNOS, inducible iNOS, and endothelial eNOS. H_2_S is produced by cystathionine *β*-synthase, cystathionine *γ*-lyase, L-cysteine desulfhydrase, and 3-mercaptopyruvate sulfurtransferase (for an overview see [1] and references within). In addition to the production, the degradation of these molecules is also enzymatically regulated (Figure 2()). Contrary to previous understanding, free oxygen and nitrogen species cannot generally oxidize thiol groups directly. The reaction rate of H_2_O_2_ with the highly abundant peroxidases of the Trx family, Prxs, ranges from 10^6^ to 10^8^ M^−1^·s^−1^. The reaction rate of other reactive protein thiols and free Cys is significantly lower in a range of approximately 10^1^ M^−1^·s^−1^ [7, 8]. Due to high protein expression and reactivity, a molecule of H_2_O_2_ is more prone to oxidize a Prx molecule than the thiol group of any other protein. Prxs are peroxidases that can function in cellular signaling as peroxide sensors. Moreover, H_2_O_2_ signaling can be conducted via GPxs and GSH [9]. Trx family proteins are key regulators of redox signaling by regulating the redox state of particular substrate proteins. They catalyze disulfide reduction and isomerisation reactions and regulate deglutathionylation, as well as denitrosylation and depersulfidation. Moreover, they are also involved in the oxidation of thiols, for example, by catalyzing S-glutathionylation, transnitrosylation, and S-sulfhydration. Trx proteins contain the structural Trx fold and an active site motif that contains one or two cysteinyl residues and is essential for the catalytic monothiol and dithiol mechanisms. Substrates of Trx family proteins include enzymes such as ribonucleotide reductase [10, 11] Sirtuin-1 [12], caspase-3 [13], the mitogen-activated protein (MAP) kinase apoptosis signal-regulating kinase 1 (ASK1) [14] and mercaptopyruvate sulfur transferase (MST) [15], transcription factors such as nuclear factor kappa B (NF*κ*B) [16], and signal transducer and activator of transcription 3 (STAT3) [17]. Moreover, components of the Wnt signaling pathway (dishevelled [18]), cytoskeletal dynamics (e.g., collapsin response mediator protein 2 [19, 20]), and innate immunity (e.g., myeloid differentiation primary response 88 (Myd88) [21] and a disintegrin and metalloproteinase 17 (ADAM17) [22]) are regulated by Trx proteins. So far, not much is known about the specificity of substrate recognition. However, it is known that not every surface-exposed Cys residue is involved in redox regulation. Lillig and Berndt have shown that the reactivity of a cysteinyl residue depends on the surrounding amino acids creating the electrostatic and hydrophobic environment of the thiol group [23]. Recently, it was demonstrated that substrate recognition depends on kinetic constraints, complementary molecular geometries, and the electrostatic surface potential of the oxidoreductase and the target protein [8, 24].

## 2. Redox Regulation of the Inflammatory Response

Upon tissue damage and infection, the inflammatory response is induced. This highly regulated and protective process facilitates the removal of foreign and/or damaged components, as well as tissue repair and is terminated when a return to physiological conditions is achieved. The inflammatory response is composed of distinct receptor proteins, inflammatory mediators, and specialized cell types, as well as changes in tissue homeostasis and blood flow. Initiation of inflammation is reliant on the production of a number of cytokines which are produced by activated cells of the innate immune system in response to a range of stimuli. Proinflammatory cytokines are essential for the activation of the adaptive immunity, that is, B- and T-lymphocytes. In some circumstances, the production of these proinflammatory cytokines is maintained beyond that required to facilitate microbial destruction and tissue repair, resulting in a chronic inflammatory response where both innate and adaptive immune cells are chronically activated, inducing tissue damage and subsequent autoimmune disease. Even though the exact redox signaling cascades are not fully understood, it is well known that the production of reactive oxygen species (ROS) and reactive nitrogen species (RNS) is essential for the onset, progression, and also the termination of inflammatory processes. Redox-regulated processes involve the innate, as well as the adaptive immunity, for example, the oxidative burst of immune cells and pathogen killing, cellular signal transduction, and regulation of gene transcription, cytokine release, and antigen presentation as well as the regulation of the activation, differentiation, and migration of immune cells and wound healing [1, 25–27]. Particularly, not only NO and H_2_O_2_ are essential during inflammation but also H_2_S has been shown to possess anti- and proinflammatory functions [28, 29]. Production of NO as a signaling molecule with microbicidal, antiviral, and antiparasital as well as immunomodulatory functions is essential for inflammatory processes (reviewed in [30, 31]). NO constitutes an important second messenger in the inflammatory response with various functions in the classical activation during the onset of the inflammation, signal transduction, revascularisation, and tissue repair [32].

**Figure 3:**
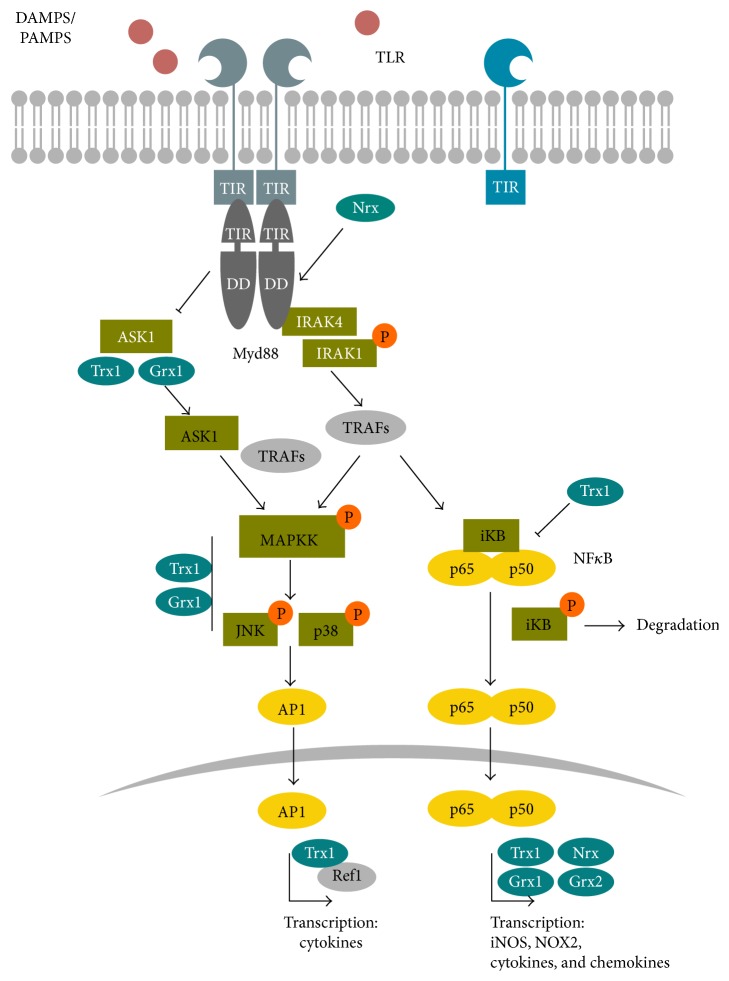
TLR signaling is redox-regulated. The general concept of the TLR signaling is illustrated, emphasizing the redox-regulated steps and molecules; note that this illustration is simplified and that specific TLR pathways include different proteins. PAMPs and DAMPs are recognized by their specific TLR, which can lead to homo- and heterodimerisation. Upon ligand binding, the TLR associates with the adaptor protein Myd88, which is sensitive to oxidation by hydrogen peroxide and can be regulated by Nrx. Myd88 recruits IRAK4 that phosphorylates IRAK1, which in turn activates additional proteins (e.g., TRAFs and IKK, not shown). MAP kinases and NF*κ*B are activated. MAP kinase signaling is regulated by Trx1 and Grx1 and eventually activates the transcription factor AP1, which has two Cys residues in its DNA binding domain that are reduced by Trx1 via Ref1. The NF*κ*B subunits p50 and p60 are kept in an inhibitory i*κ*B/NF*κ*B-complex in the cytosol. Reduced Trx1 inhibits the dissociation of this complex. Upon dissociation, i*κ*B is phosphorylated and degraded by the proteasome. NF-*κ*B translocates into the nucleus, where it binds to the DNA, a process that depends on the reduction of Cys62 and is regulated by Trx1, Grx1, and/or Nrx. An additional redox-regulated pathway involving ASK1 exists in TLR4 signaling. ASK1 is kept in an inactive complex by reduced Trx1. Upon TLR activation, Trx1 is oxidized, the complex dissociates and active ASK1 regulates JNK activity via different proteins including TRAFs.

Reactive species are produced by phagocytic cells of the innate immune system, such as monocytes, macrophages, neutrophils, and dendritic cells, during the oxidative burst in order to kill pathogens as well as during tissue repair [33, 34]. Myeloperoxidase (MPO) catalyzes the reaction of H_2_O_2_ to highly oxidizing and microbicidal hypochlorous acid (HOCl) and hypobromous acid. This reaction can also be catalyzed by the eosinophil peroxidase. Another bactericidal and fungicidal enzyme that acts in the innate immune defence is the heme protein lactoperoxidase (LPO) generating hypothiocyanite (^−^OSCN) from thiocyanate (SCN^−^) and H_2_O_2_. The latter is a downstream metabolite of superoxide that is enzymatically produced by the NOX enzymes Duox1 and particularly Duox2 [35, 36]. In addition, NO and further RNS have been shown to be present in the phagosome and participate in eradication of pathogens [32]. Activation of NOX and the oxidative burst occurs only upon full activation of neutrophils in the presence of pathogens. Their antimicrobial activity can be primed by inflammatory cytokines, chemokines, anaphylatoxins, or pathogen-associated molecular pattern (PAMPs), for example, compounds of bacterial cell walls such as lipopolysaccharides (LPS) and lipoteichoic acid, flagellin, and bacterial DNA that are recognized by pathogen recognition receptors such as Toll-like receptors (TLRs) and cytoplasmic NOD-like receptors (NLRs) [37]. The latter are also part of the inflammasome that facilitates the cytosolic, caspase-1-mediated maturation of inflammatory cytokines and that has been shown to be redox-regulated [38], (reviewed in [26]). Both, NLRs and TLRs also recognize endogenous damage-associated molecular patterns (DAMPs), such as the redox-regulated high mobility group protein 1 (HMGB1) or metabolites like ATP, which are also known as danger signals [39]. TLRs are not exclusively expressed in phagocytic cells and are present in the first barriers of defence, such as the skin, airway, blood vessels, and colon. TLRs are involved in ROS production. Interestingly, LPS-activated neutrophils produce H_2_O_2_ that induces the TLR2 expression in endothelial cells promoting the immune defence via redox-regulated signaling events [40]. The cytosolic Toll/IL-1 receptor (TIR) domain of TLRs associates with the signal transduction adaptor protein Myd88 that recruits and activates a set of proteins, inducing downstream Map kinases (e.g., JNK and p38) and the phosphorylation and degradation of I*κ*B, NF*κ*B activation, and expression of target genes (Figure 3()) [41]. Various components of this pathway are susceptible to redox regulation and were shown to interact with Trx family proteins, including NF*κ*B, the transcription factor that controls, for example, the expression of proinflammatory cytokines, chemokines, growth factors, prostaglandins, adhesion molecules, and NOX2 as well as iNOS and also nNOS [1, 42, 43], promoting leukocyte recruitment and activation of the surrounding tissue. Interestingly, cytokines can be expressed as cytosolic or membrane-bound “precursors” and are activated and released by redox-regulated, proteolytical cleavage via cytosolic multiprotein complexes called inflammasomes or specific proteases such as ADAM17 [26, 44–47]. Cytokines are not the only proteins that are secreted upon inflammation. A large number of proteins secreted from innate immune cells in response to inflammatory stimuli have been shown to be glutathionylated [48]. Recent studies have seen the refinement of redox proteomic techniques to interrogate those proteins, identifying a substantial number of glutathionylated proteins, both intracellular and secreted [49, 50]. Among the secreted proteins, Trx1, Trx80, Prx1, and Prx2 were detected that have cytokine and/or chemokine-like functions [1, 51]. Secreted, glutathionylated Prx2 was recently described to function as danger signal [52]. And also, the related macrophage-inhibitory factor (MIF-1) has immunomodulatory functions [1].

Redox regulation of inflammation and of immune responses is not restricted to the activation and subsequent activity of innate immune cells. Generation of both humoral and cell-mediated adaptive immunity depends on activation of T helper cells, a process heavily reliant on the redox potential of the microenvironment of these cells [53, 54]. A reducing environment is necessary for both optimal activation of T-cells [55, 56] and for the downstream proliferation of these cells [57, 58] that is essential for generating an adaptive immune response. As these effector CD4^+^ T-cells are essential for inducing full activation and class switching in activated B lymphocytes, the effects of changes in the redox environment also extend to the humoral arm of the adaptive immune response. It is perhaps unsurprising that redox changes in antigen-presenting cells can also help to determine whether T-cells develop into Th1 or Th2 cells [59, 60] given the importance of the interactions between T-cells and their antigen-presenting cells in T-cell activation. Increases in cellular ROS levels have been shown to be essential, for example, during T-cell activation, antigen presentation, and receptor-mediated cell signaling. Interestingly, administration of antioxidants such as the seleno-compound ebselen inhibits and impairs these functions [1, 61]. This may help to explain observations that autoimmune diseases such as rheumatoid arthritis [62] and multiple sclerosis [63] are associated with increased levels of oxidative stress. Like many autoimmune diseases and chronic inflammatory diseases, it is still unclear whether oxidative stress is a cause or effect of these conditions. However, for these particular conditions, treatment with antioxidants does actually ameliorate disease, at least in animal models [64], suggesting that oxidative stress does indeed play a role. Furthermore, one of the frontline treatments for people with multiple sclerosis, dimethyl fumarate, exerts its therapeutic effects by upregulating antioxidant enzyme synthesis [65]. One possible mechanism by which oxidative stress could impact these conditions is via effects on T-cells that infiltrate the sites of disease, a recognized phenomenon in these pathologies [66]. If these cells then encounter relatively oxidizing conditions, this could influence their activation into the more inflammatory phenotypes such as Th1 and Th17 phenotypes, thereby exacerbating disease. Indeed, it has been suggested that exposure of T-cells to increased oxidative stress in rheumatoid arthritis causes them to become refractory to apoptosis leading to a perpetual immune response [67].

Within the next chapter, we will introduce distinct thiol switches and their impact on cell signaling and inflammatory processes (Table 1).

## 3. Thiol Switches in the Inflammatory Response

### 3.1. TLR Signaling

**Figure 4:**
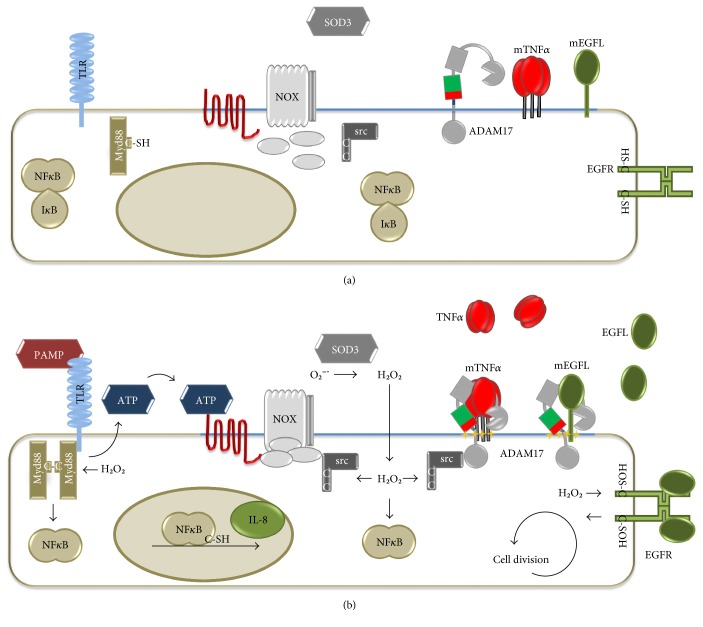
Pathogen detection and ROS-dependent defence and regeneration mechanisms. Epithelial cells are constantly exposed to pathogens. The redox state, the localisation, and the activity of different molecules and proteins are altered in the absence (a) or in the presence (b) of pathogens. Activation of TLRs by PAMPs and Myd88 recruitment induce secretion of ATP, which functions as danger signal and activates NOX. TLR and NOX activation both result in NF*κ*B activation, via Myd88 or src, respectively. NF*κ*B translocates to the nucleus and induces the expression of, for example, chemokines such as IL-8, promoting leukocyte recruitment. Myd88 dimerizes upon H_2_O_2_ exposure forming disulfide bridges. Src oxidation stabilizes the active conformation of the protease and the oxidation of cysteine residues near the ATP-binding site of the EGFR enhances its activity. Extracellular ATP leads to the activation of the shedding activity of ADAM17. ADAM17 releases soluble TNF*α* and ligands of the EGFR, such as TGF*α* and HB-EGF, from the cell surface, whereas TNF*α* promotes inflammation; signaling via the EGFR leads to regeneration due to induction of cell growth and division (mTNF*α*: membrane-bound TNF*α*; mEGFRL: membrane-bound EGFR ligands).

In terms of redox signaling, the production of the second messenger H_2_O_2_ is closely linked to the transmembrane multidomain NOX complexes. These transport electrons via NADPH, flavin-adenine dinucleotide (FAD), and heme from the cytoplasmic side of the plasma membrane to the extracellular part, where they are transferred to oxygen. By the action of extracellular SOD3, the produced superoxide is converted to H_2_O_2_, which passes the membrane by diffusion or via aquaporins (Figure 2). Superoxide/H_2_O_2_ production occurs in close proximity to the receptor complex, potentially in specific signaling platforms within lipid rafts, caveolae, or endosomes [68]. The NOX family comprises seven members, NOX1 to NOX5 and Duox1 and Duox2. The structure and regulation of the different NOXs have been extensively reviewed previously [69–72]. NOX-dependent ROS production can depend on endocytosis of activated receptor NOX complexes in redox-active endosomes, the redoxosomes. The formation of redoxosomes occurs out of lipid rafts, which contain inactive NOX as well as ligand-bound receptors that initiate NOX activity and require activated Rac1. Inhibition of endocytosis and formation of redoxosomes reduces superoxide formation and downstream activation of NF*κ*B. For proper signaling, SOD activity and chloride channels are required, which are believed to export superoxide into the cytoplasm and import protons that stabilize the pH within the redoxosomes (reviewed in [73, 74]). Interestingly, this was demonstrated for IL-1*β*- and TNF*α*-induced signaling, but not for thrombin-activated NOX1 [74–77]. NOX1 is expressed in the colon and the vascular system and can be triggered by flagellin, via TLR5 [78], by LPS via TLR4 [79], and by CpG oligonucleotides via TLR9 [80] and is sensitive to IFN*γ* [81]. NOX2 constitutes the first identified NOX, which is highly expressed in phagocytic active neutrophils and macrophages and to a much lower rate in dendritic cells [82]. NOX2 is sensitive to multiple TLRs [83] and essential for the oxidative burst. The assembly and activation of NOX2 occur upon fully activation of neutrophils in the presence of pathogens. Dendritic cells are specialized for antigen presentation, and NOX2 is needed for proper antigen presentation towards T-cells [84, 85]. In the airway epithelium, Duox1 was shown to depend on TLR4 [86]; regulating the expression of chemokines, which attract neutrophils and macrophages [83, 86, 87]. The physical interaction between the TIR domain of TLR4 and the cytoplasmic tail of NOX4 results in an activation of src, which phosphorylates IkB*α*, thereby activating NF*κ*B and target genes [87]. The activity of src is regulated by tyrosine phosphorylation and can be boosted by a thiol switch [88]. Protein tyrosine phosphatases (PTPs) remove an inhibitory phosphorylation of a C-terminal Y527 residue and thus its inhibitory interaction with the SH2 domain of the kinase, followed by autophosphorylation. This active conformation of the protein is stabilized by a reversible thiol switch. C245 and C487 are oxidized and form a disulfide bond connecting the SH2 and the kinase domain. An exchange of these cysteinyl residues to alanine residues results in a redox unresponsive variant [88, 89]. Interestingly, src is not only involved in the regulation of NOX signaling but also targets the epidermal growth factor receptor (EGFR) that was also shown to undergo thiol oxidation. The targeted cysteine residue is located close to the ATP-binding site within the cytoplasmic part of the receptor protein (Figure 4). An exchange of the cysteine residue to a serine residue induces a 2.5-fold increase in the ATPase activity of EGFR [90, 91]. Besides src and EGFR, PTPs, for example, PTP1B, are targets for redox modification, that is, reversible oxidation of the catalytic active cysteine that renders the protein inactive [91, 92]. All three proteins are targeted by H_2_O_2_, produced by Duox1 in response to extracellular ATP, which functions as danger signal in the airway epithelium host defence [91]. These three examples show how specific and diverse redox regulation can occur during the same conditions and stimuli within a signaling cascade. Even though all transducers are oxidized at one or two particular Cys residues, the effect on the protein activity differs from being turned on or off like a redox switch to being modulated! Even though the oxidation has been shown, the exact regulatory mechanisms are still mostly elusive. It is however tempting to speculate that the oxidation by hydrogen peroxide is mediated via cytosolic Prxs and the reduction via, for example, Trx. Trx proteins have been already shown to regulate Myd88 and downstream Map kinases. Most TLRs need the adaptor protein Myd88 for signal transduction, which functions downstream of the signal-receptor complex upon ligand binding. Myd88 oligomerizes with the interleukin-1 receptor-associated kinase (IRAK) forming a signal initiation complex. The complex signal transduction involves various proteins and kinases, eventually triggering MAP kinases and NF*κ*B signaling pathways (Figure 3) [41]. Recently, Stottmeier and Dick demonstrated that Myd88 undergoes redox regulation. In the presence of H_2_O_2_, Myd88 dimerizes and forms disulfide-linked conjugates with other proteins via eight conserved Cys residues (Figure 4). Interestingly, the oxidation by hydrogen peroxide is comparably sensitive to oxidation of Prx2 [93]. S-Nitrosylation of distinct Cys residues of Myd88 has also been described [94]. Nucleoredoxin (Nrx) controls TLR4 signaling by regulation of Myd88, that is, by stabilizing the interaction of Myd88 with flightless homolog 1 [21]. Moreover, Nrx was shown to regulate the adaptor protein, potentially as a disulfide reductase. Nrx is related with Trx, which additionally catalyzes de- and transnitrosylation of proteins. It is tempting to speculate that Nrx has similar catalytic mechanisms and that it could regulate Myd88 activity not only as disulfide reductase but also by regulating S-nitrosylation. Interestingly, different regulatory functions for the eight Cys residues have been introduced. Mutation of C113 inhibited NF*κ*B signaling, whereas mutating the other Cys residues individually and especially simultaneously enhanced NF*κ*B activity. Note that these seven Cys residues are all located in the TIR domain [93]. Different kinases, including the MAP kinases, are responsible for signal transduction and have been described to be susceptible to redox regulation. Trx1 and also Grx1 regulate ASK1 and downstream kinases such as ERK, JNK, and p38. The reduced oxidoreductases bind to ASK1 and thereby inhibit the enzymatic activity of the kinase. In case of Trx1, the protein interaction initiates ubiquitin-mediated degradation. Oxidation of the oxidoreductases induces the dissociation of the complex and restores kinase activity [1, 14, 95, 96]. Interestingly, ASK1 is involved in TLR4 signaling and has however not been shown to be essential for other TLR pathways (Figure 3) [97, 98].

Following the cascade of cell signaling-transducing molecules, effector molecules are also posttranslationally modified, for example, the transcription factor NF*κ*B, which is highly regulated (for an overview see [36]). Comparable to other transcription factors such as AP1 and HIF1*α*, the DNA binding of NF*κ*B is regulated by specific Cys residues that are susceptible to oxidation. The NF*κ*B subunit p50 contains a cysteine residue in position 62 that promotes DNA binding in its reduced form. Alkylation, oxidation, or mutation to Ser or Ala of that particular cysteine inhibit DNA binding. It was shown that Cys62 can undergo S-glutathionylation and can also form a sulfenic acid [99]. Interestingly, various members of the Trx family have been shown to be involved in NF*κ*B regulation. Even though it was shown that NF*κ*B is a substrate for Trx1, Grx1, Grx2, and Nrx, the physiological impact during cellular signaling is poorly understood [1, 16, 100, 101]. Overexpression of Grx3 in T-cells on the other hand inhibited NF*κ*B- as well as AP1-induced gene expression [102]. Besides the DNA binding, the nuclear translocation is also redox-regulated. Reduced Trx1 inhibits the dissociation of the inhibitory i*κ*B/NF*κ*B complex. Upon dissociation of the complex, i*κ*B becomes phosphorylated and degraded by the proteasome. NF*κ*B translocates into the nucleus (Figure 3). Apart from the regulation of transcription factors, gene expression can also be redox-regulated by, for example, the nuclear histone deacetylase and thus by chromatin remodelling [103].

### 3.2. Redox Regulation of Inflammatory Mediators

#### 3.2.1. The NLRP3 Inflammasome Is Redox-Regulated

ROS were shown to control the NLRP3 inflammasome, a multiprotein complex that transfers the precursor of IL-1*β* in its mature and active form [26]. This process was shown to be regulated via Trx1. The cytosolic oxidoreductase binds thioredoxin-interacting protein (Txnip), a protein that was suggested to act as an endogenous inhibitor of Trx [104]. In this complex, Txnip is not able to interact with and activate NLRP3. Upon oxidation of Trx1, the Trx1-Txnip complex dissociates and Txnip binds to NLRP3. Other mechanisms have been proposed in the regulation of the NLRP3 that is activated by various different stimuli, which are redox-independent or might depend on the redox regulation by Trx1 and Txnip [44].

#### 3.2.2. Ectodomain Shedding by ADAM17—A Regulatory Thiol Switch in ADAM17 in Inflammation and Tissue Regeneration

**Figure 5:**
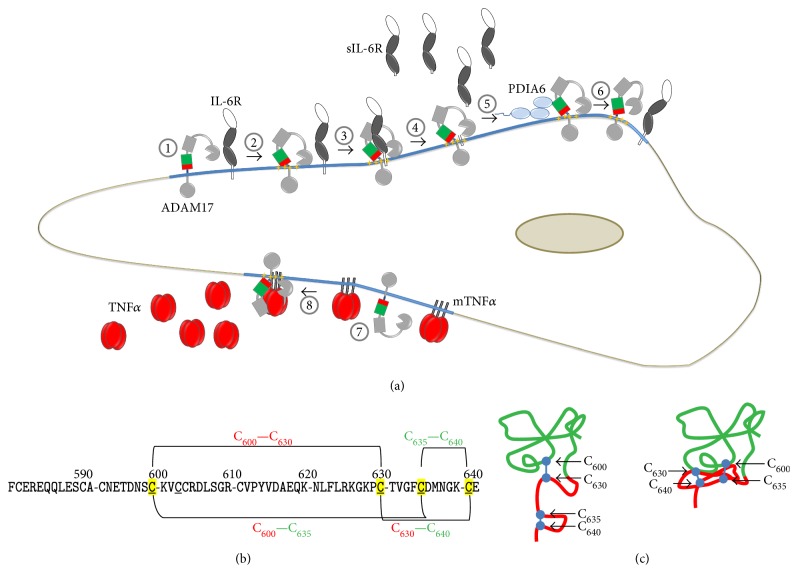
Thiol switch in ADAM17. (a) (1) ADAM17 is active within lipid rafts (blue line). (2) Different stimuli induce the exposure of phosphatidylserine (yellow stars), that interacts with the open and active conformation of the MPD. (3) This process allows ADAM17 to bind and (4) release substrates from the cell surface, for example, soluble interleukin 6 (sIL-6R). (5) Reduced extracellular protein disulfide isomerase PDIA6 catalyzes the disulfide isomerisation targeting the open MPD. (6) The resulting close and inactive structure of ADAM17 is not able to bind and process its substrates. (7) Membrane bound TNF*α* (mTNF*α*) is another substrate of ADAM17, (8) which is released upon activation of ADAM17 and also promotes immune response and inflammation. (b) Primary structure of the MPD of human ADAM17, indicating the disulfide bridges involved in the thiol switch. The linear pattern (C_600_–C_630_, C_635_–C_640_) constitutes the active, the overlaying pattern (C_600_–C_635_, C_630_–C_640_), the inactive conformation. (c) Structural consequence of the thiol switch of ADAM17. The red-colored part is highly flexible in the open MPD and therefore not visible in the NMR data. The right structure represents the closed conformation of ADAM17 solved by NMR, in which the red part is packed tightly to the upper, green colored part of the MPD.

Phagocytes release various proinflammatory mediators to promote leukocyte recruitment and activation of the surrounding tissue. In this process, the IL-6R and the membrane-bound precursor of TNF*α* are proteolytically cleaved by ADAM17; this shedding process leads to the generation of proinflammatory acting TNF*α* and sIL-6R (Figure 5()). Shedding of IL-6R from apoptotic neutrophils generates an agonist of IL-6 signaling, allowing the activation of cells, which do not express the membrane-bound IL-6R, but the ubiquitously expressed signaling subunits of the IL-6 receptor complex gp130. This transsignaling mechanism promotes the attraction of monocytic cells and inflammation [46, 105, 106]. Moreover, ADAM17 cleaves members of the EGFR ligand family, which are essential for their function as growth factor and tissue regeneration [107–109]. Various ligands of the TLR and NOXs induce the activity of ADAM17 that is essential for immune response/inflammation and regeneration (Figure 4()) [47, 83, 110, 111]. In the healthy airway, TLR signaling can be upstream of exogenous ATP [112, 113]. Duox1 is recruited to ATP-activated purinergic P2YR, followed by association with src, which becomes oxidized. Src in turn oxidizes and activates ADAM17, which amplifies EGFR activation and promotes immune defence and regeneration, involving an ERK1/2-dependent production of the neutrophil attractant IL-8 (Figure 4()) [114, 115]. Dysregulation of this pathway has been linked to inflammatory diseases, for example, cystic fibrosis and chronic inflammatory airway disease [116–120]. LPS-induced activation of ADAM17 in macrophages was shown to rely on the activity of PKC*δ* and p38. This activation is TLR4- and NOX2-dependent and targets the tyrosine kinase Mer, which inhibits inflammatory signaling during efferocytosis [121]. In primary monocytes, LPS-induced activation of ADAM17 is also mediated by ROS and p38 [122]. In hepatocytes, src activates NOX1, which in turn activates ADAM17 that releases TGF*α* for the stimulation of the EGFR [123]. This process is caveolin-1-dependent. ADAM17, NOX1, and NOX2 are located and active within lipid rafts [75, 123–126]. The interaction of NOX1 and ADAM17 was shown by coimmunoprecipitation [127]. Interestingly, ADAM17 can also be activated by mitochondrial ROS in a src- and PKC-independent way via the activation of the P2Y receptor by ATP in fibroblast [128] and FAS-mediated apoptosis in neutrophils [129]. The activation of ADAM17 by members of the NOX family appears to be dependent on the activity of kinases such as src, PKC, p38, and/or ERK1/2. These kinases have been previously shown to be involved in the regulation of ADAM17, which is multilayered and only partially understood [130–133]. Interestingly, the extracellular part of ADAM17 is a target for regulatory events. In its mature form, the N-terminal catalytic domain is followed by a disintegrin domain, a membrane-proximal domain (MPD), and a conserved helical stalk region called conserved ADAM seventeen interaction sequence (CANDIS), a single transmembrane region and a cytoplasmic tail [134–137]. The MPD exists in two conformations that control the activity of the protease [135, 138]. A linear order of two disulfide bridges (C_600_–C_630_ and C_635_–C_640_) leads to an open, flexible structure, which is able to interact with the plasma membrane and substrates [139, 140]. Reduced protein disulfide isomerase (PDI), a member of the Trx protein family, catalyzes the isomerisation to an overlaying pattern (C_600_–C_635_ and C_630_–C_640_) causing a close, compact structure, which abrogates membrane binding and substrate recognition and thereby ADAM17 activity. In line, PDIA1 and PDIA6 were found to act as negative regulators [22, 135, 141] (Figure 5()). The thiol switch as a general posttranslational mechanism to regulate the activity of members of the ADAM family appears to be unlikely since ADAM17 and its closest relative ADAM10 are atypical members of the protein family. The other members lack the redox-regulated MPD domain and contain a cysteine-rich and an EGF-like domain instead [134, 136]. Therefore, no comparable posttranslational thiol switch can be expected and indeed so far no posttranslational regulation of these proteases via NOX, ROS in general or specific oxidoreductases has been described to our knowledge. It is however possible that the activity of ADAM10, which contains a MPD homolog to the one of ADAM17, is regulated via a comparable thiol switch. The isolated open form of the ADAM17-MPD can be expressed as a soluble protein and the closed from can be obtained by refolding or by enzymatic catalysis by PDIs, converting the open form to the close form. So far, no open ADAM10-MPD was obtained by expression in *E. coli* (unpublished observations), indicating that no open form exists and/or that the interaction with the N-terminal located disintegrin domain might be tighter and more important for the stabilization than in ADAM17. This might point against a regulatory thiol switch of ADAM10 and fit to the observation that the activity of ADAM17 is more strongly regulated than the activity of ADAM10, which can be constitutively active. However, a thiol switch in ADAM10 cannot be excluded since reports indicate that the shedding activity of ADAM10 can indeed be stimulated by ROS [142, 143]. PDIs attack the CKVC motive in the MPD of ADAM17, which is evolutionarily conserved in vertebrates, but not present in animals such as pike, hamadryad, or drosophila. ADAM10 on the other hand contains the CHVC motif that is also conserved in evolutionary higher animals. This indicates that during evolution with increased complexity and potential higher risks of uncontrolled substrate release, a regulatory mechanism of the protease became essential. Note that the posttranslational regulation of proteins by a thiol switch in their ectodomains is not unique for metalloproteases. For example, CD30 contains no CKVC or CHVC motive and is targeted by Trx1 which results in an altered ligand binding [144], whereas ADAM17 becomes inactivated by the thiol switch, and *β*1 and *β*3 integrins become activated [145]. Intriguingly, this can be catalyzed by identical PDIs, such as PDIA1 and PDIA6. Since *β*1 and *β*3 integrins contain numerous CXXC motives, but not a CKVC motive, PDIs may recognize different CXXC motives.

#### 3.2.3. The Immunomodulatory Functions of HMGB1 Are Regulated via Three Cys Residues

HMGB1 comprises the HMG A box essential for DNA binding, the HMG B box essential for DNA binding and proinflammatory functions (i.e., amino acids 89 to 108), and an acidic C-terminus [146]. HMGB1 conducts various functions depending on its localization. Nuclear HMGB1 is, for instance, involved in DNA organization and gene transcription; cytosolic HMGB1 regulates the inflammasome, pyroptosis, and the autophagy/apoptosis balance; and extracellular HMGB1 has been described as one of the first DAMPs with proinflammatory activities in distinct cell culture and animal models, as well as in patients suffering from sterile or infectious inflammation (reviewed in [147]). LPS-stimulated monocytes secrete HMGB1 nonclassically via exocytosis of secretory lysosomes induced by lysophosphatidylcholine that is produced comparably late during inflammation [148]. Interestingly, oleanolic acid is a natural inhibitor of HMGB1 release by LPS-stimulated RAW264.7 macrophages. Even though the exact mechanism is not fully understood, it involves the activation of Nrf2 that binds to the ARE of heme-oxygenase-1 [149]. HMGB1 is also released during necrosis or cell damage, however, not during apoptosis [150]. HMGB1 leakage has also been associated with high levels of superoxide and peroxynitrite [151]. HMGB1 has three Cys residues in the positions 23, 45, and 106. We have recently shown that TNF*α*-induced HMGB1 secretion from HEK293 cells does not depend on the redox state of the protein [51]. Note that the translocation from the nucleus to the cytosol depends on posttranslational modifications such as acetylation and potentially also thiol oxidation [152, 153]. Especially, the substrate interaction and the distinct functions of HMGB1 are redox-regulated. An intramolecular disulfide between Cys23 and Cys45, as well as the reduced Cys106, located in the HMG B box, is essential for TLR4/MD2 binding, macrophage activation, and cytokine release. Fully oxidized, that is, three sulfonates and fully reduced HMGB1 do not affect TLR4 signaling [154, 155]. However, the latter shows chemotactic activity by interacting with the chemokine CXCL12 that binds to the chemokine receptor CXCR4. Interestingly, a redox-inactive mutant, containing three Ser residues instead of Cys residues, is even more active in terms of leukocyte recruitment than the fully reduced protein [151, 156, 157]. Even though the redox state of the protein has been linked to particular substrates and functions in different compartments, the regulation of the thiol switches of HMGB1 has not been fully understood. It is however clear that these switches constitute physiological mechanisms to regulate and modulate the inflammatory activities of the protein. Interestingly, HMGB1 was shown to interact with the oxidoreductase glutaredoxin [153] and also Trx1 was shown to be able to reduce the intramolecular disulfide [153, 158].

#### 3.2.4. Extracellular Redoxins Act as Immune Mediators

Distinct members of the Trx family of proteins have been described to be secreted in various cell and animal models, as well as in patients suffering, for example, from inflammatory diseases (reviewed in [1]). Trx1 was originally known as T-cell leukemia-derived factor that was shown to induce the IL2 receptor [159] and the expression of various cytokines [160]. In addition, the truncated Trx80, formerly characterized as eosinophil cytotoxicity-enhancing factor, has been shown to be secreted functioning as cyto- and chemokine [161]. Apart from its cytokine and chemoattractant functions, there are also controversial findings that imply an anti-inflammatory role. One potential mechanism could involve the regulation of the proinflammatory macrophage migration inhibitor factor (MIF). Interestingly, MIF also belongs to the Trx family of proteins and is involved in the innate immune response [162]. Prx2 is a highly expressed intracellular peroxidase that is released from myeloid cells in response to inflammatory stimuli. Once released from cells, Prx2 has proinflammatory activity, essentially behaving as a DAMP [52]. Intriguingly, the release of Prx2 from cells under inflammatory conditions is mediated by two types of thiol modifications involving all three cysteine residues. Prx2 is released from LPS-stimulated mouse macrophages in a glutathionylated form [52]. A second thiol redox change involves oxidation of two cysteine residues forming a disulfide bond, which induces protein dimerization and results in its release from the cell via exosomes [51]. Mutation of either one of the Cys residues involved in the disulfide bridge prevents secretion of the enzyme. Recombinant Prx2 is able to stimulate the release of TNF*α* from both mouse macrophages and primary human monocytes [51, 52]. Prx1 is also released from mouse macrophages in response to LPS. It was detected in the secretome of LPS-stimulated cells in a glutathionylated form and also exhibits the reliance on Cys oxidation for the release from cells. Thus, it appears that redox modulation regulates the release of these enzymes from cells contributing to the local inflammatory response. In addition, redox changes provide a novel mechanism by which proteins are processed for export from cells during inflammation, at least for Prx1 and 2. As such, there is the potential for the development of novel therapeutic strategies for modulating the redox environment in order to dampen the inflammatory response. Note that also Trx1 was detected in the proteomic analysis.

## 4. Clinical Significance

Biomarkers for inflammatory disorders include oxidative modifications of DNA, proteins, and lipids and have been reviewed in [25]. Even though the redox state of particular proteins is not easily accessible in patient material due to a general lack of specific tools, the expression, localization, and activity of redox enzymes, for example, Trx family proteins have been studied in various diseases (Table 2) [1]. Moreover, different redox enzymes have been identified as potential targets for therapy in a number of diseases, including inflammatory disorders. The neutrophil-derived myeloperoxidase is known as one of the most potent oxidant-producing proteins. Increased MPO activity and excessive production of hypochlorous acid contribute to chronic inflammation and organ damage in many tissues [163, 164]. Elevated expression was described in cardiovascular disease [165, 166], presumably due to its oxidation of low- and high-density lipoprotein [167], as well as rheumatoid arthritis [168]. MPO also seems to be a risk factor in heart failure and acute coronary syndrome [169]. In tracheal aspirates, elevated levels of chlorinated proteins, trace markers of MPO activity, are believed to contribute to chronic lung infection in infants [170]. Accordingly, many studies have been conducted in search of nontoxic, reversible MPO inhibitors preferably binding the native protein [171–173]. Interestingly, neutrophil extracellular traps are decorated with active MPO [174] and are associated with chronic inflammation in many diseases too [175]. Neuron-derived MPO seems to contribute to Alzheimer's disease, a neurodegenerative disorder that has also been linked to neuroinflammation [176]. It is worth mentioning that elevated MPO activity is associated with an overall better outcome in specific cancer chemotherapy [177]. However, MPO is tightly linked to many clinical observations but redox signaling pathways beyond localized HOCl-mediated oxidation remain to be studied in most pathologies.

The heme protein lactoperoxidase is found in secretion liquids such as tears, milk, and saliva [178]. Saliva in particular has been thoroughly investigated in different oral diseases. The effect of orally administered LPO was weak on periodontitis and bacteriological profile [179]. However, LPO activity itself seems to be increased in periodontitis [180] although thiocyanate is not increased in this disease [181]. There is no association between recurrent aphthous stomatitis and salivary thiocyanate levels [182] but patients with aphtous ulcers have significantly lower oral LPO levels [183]. Xylitol increases oral LPO activity but not thiocyanate levels, and this may account for the cariostatic effect of xylitol. Also, compounds with a 3,4-dihydroxyphenyl structure significantly enhance LPO activity [184] but the clinical implication of this finding remains to be elucidated. Frequent tobacco consumption puts people at risk for oral cancer [185]. Saliva levels of thiocyanate are strongly increased in smokers [186] whereas LPO activity is blocked by tobacco smoke [187]. Whether LPO is crucial in oral carcinogenesis currently remains unknown.

The seven NOX members generate superoxide and secondarily H_2_O_2_. In chronic granulomatous disease, that is, a group of hereditary defects that result in an increased susceptibility to various bacterial and fungal infections, a functional NOX attenuation leads to life-threatening infections [188]. Hereby, the degree of attenuation governs patient prognosis [189]. Genetic defects in components of NOX2 have been linked to chronic granulomatous disease [69, 84, 190]. NOX proteins have been associated with cardiovascular risk factors contributing to atherosclerosis, vascular dysfunction, hypertension, vascular hypertrophy, and thrombosis [191]. An upregulation of NOX2 was detected upon myocardial infarct in cardiomyocytes [192] and in failing, however not in nonfailing hearts [193] as well as in saphenous veins of patients with heart failure [194]. NOX2-enriched veins may contribute to endothelial dysfunction [195]. Accordingly, targeting NAPDH oxidases in cardiovascular disease was suggested to be of clinical benefit [196]. NOX can be activated in the blood vessel walls via angiotensin II [197] causing cardiovascular disease [198]. NOX is also a target in diabetic nephropathy [199], and an orally administrable inhibitor (GKT137831) has completed phase 2 trial (NCT02010242) but results have not yet been published. NOX1 inhibition is also a therapeutic strategy against hypertension [200] that is tested in clinical trials for cardiovascular conditions [201]. Particularly, the NOX inhibitor Dextromethorphan reduced hypertension in a multicenter trial [202]. In malignancies, NOX4 is elevated in brain, colorectal, gastric, lung, and pancreatic cancer [203]. Accordingly, NOX enzymes also constitute promising targets in cancer therapy [204]. Gentian violet, a NOX1 inhibitor, showed promising effects in the palliation of a melanoma patient [205]. Yet, NOX1 does not correlate with melanoma invasiveness [201]. This substance was also successfully used to treat the inflammatory skin condition erythema multiforme [206].

The importance of NO^.^in human health was first suggested in human ileostomy effluents showing elevated nitrite concentrations [207]. Its role in acute and chronic inflammation [208] has been investigated ever since [209]. Elevated levels of NO contribute to pathologies linked to inflammation, for example, asthma, arthritis, multiple sclerosis, transplant rejection, stroke, and neurodegenerative diseases [30, 210]. Glucocorticoids inhibit NOS [211] and thereby production of NO^•^ that has been implicated in sepsis [212]. However, clinical trials on NOS inhibition gave inconclusive results demonstrating either a negative [213], a positive [214], or no effect [215] on survival of septic patients. Short-term improvement was shown following methylene blue administration [216] whereas LNNA was ineffective [217]. NOS inhibition with L-arginine analogues such as _L_NMMA gave a more confident response with regard to cardiovascular parameters in septic patients [218]. However, the mortality rate in a phase III trial was elevated [219]. Nonetheless, this substance was shown to be effective in treating migraine attacks in a placebo-controlled clinical study [220]. Clinical trials using the NOS inhibitor GW274150 did not confirm these results, neither as early intervention [221] nor in a prophylactic therapy [222]. NOS genotype (high numbers of trinucleotides) and exhaled NO^•^ are associated with asthma [223]. The NOS inhibitor L-NIL-TA strongly reduced the amount of exhaled NO^•^ in asthmatic patients without measurable vascular side effects [224]. This finding was confirmed in another clinical trial using GW274150 with no significant improvement of the asthmatic symptoms [225]. Administration of _L_NMMA amplified bradykinin-induced asthma in volunteers [226]. GW274150 also reduced synovial joint thickness and vascularity in patients with rheumatoid arthritis [227]. In general, NOS is linked to heart disease [228]. NOS is elevated in heart tissue of patients experiencing hibernating myocardium [229], in transplanted coronary arteries [230], in rejected transplants [231], and in tissue from human heart failure [232]. NOS expression also promotes melanoma cell proliferation and is associated with poor patient survival [233]. In breast cancer [234] but not head and neck cancer [235], NOS expression corresponds to stage and invasiveness.

Oxidants have long been suggested to play a role in the central nervous system [236]. Inflammation is a key event in the onset and stage of brain disease, such as multiple sclerosis [237]. Prx1 is expressed in glial cells, whereas Prx2 expression was predominantly found in neurons [238–240]. The expression levels of both Prx1 and Prx2 are elevated in patients suffering from Alzheimer's disease [241, 242]; moreover, Prx2 and Prx6 are more oxidized in the brain [243]. Additionally, Prx2 peroxidase activity was found to be inhibited by S-nitrosylation [244] and phosphorylation [245] in Alzheimer's disease. Prx2 expression is also increased in Parkinson's disease [246, 247], whereas the Prx3 expression is decreased in the latter [248]. Prx expression is also regulated in ocular pathologies. Alongside with inflammation, Prx6 is increased in the trabecular meshwork in glaucoma patients [249] and correlates negatively with severity of cataracts [250]. Diabetic retinopathy is associated with elevated levels of Prx1 [251], with the diabetic risk being associated with increased serum concentrations of Prx4 [252]. Peroxiredoxins are regulated in cancer, a condition that heavily modulates the inflammatory environment to enhance growth [253]. Tissue and serum of lung cancer patients showed elevated levels of Prx1 and Prx3, respectively [254, 255]. Autoantibodies against Prx6 have also been shown to be of prognostic value in esophageal cancer [256]. So far, no therapeutic strategies to target Prxs were conducted.

## 5. Future Perspective

It is of great interest to understand the mechanisms of cellular signaling and how they are regulated under physiological, but generally also under pathological conditions. Even though it has been established that redox regulation and oxidative Cys modifications are essential for signal transduction and cellular processes, the identification and characterization of specific thiol switches and their enzymatic regulation constitute a big challenge in the field. Particularly, the field lacks time- and spatial-resolved *in vivo* techniques for the analysis of (i) the levels and distribution of different ROS and RNS, (ii) the particular redox state of proteins, and (iii) the impact of redox signaling on complex signaling circuits and networks. The innate and the adaptive immune responses are tightly controlled and depend on the enzymatic production of superoxide, hydrogen peroxide, hydrogen sulfide, and nitric oxide. However, not many redox-regulated protein substrates are known. Future research will identify these substrates and particular thiol switches, including intracellular, as well as membrane and extracellular proteins and the underlying regulatory mechanisms. Intriguingly, the extracellular space contains redox-active enzymes and molecules such as glutathione. It is tempting to speculate that the inflammatory response does not only constitute intracellular redox-signaling cascades but also depends on extracellular signal transduction within the microenvironment of distinct cell types.

## Figures and Tables

**Table 1:** Thiol switches in inflammatory signaling processes.

Protein	Thiol/modification	Function	Regulation	Reference
ADAM17	C_600_, C_630_, C_635_, C_640_: intermolecular disulfides	Linear order of disulfides (C_600–630_; C_635–640_): open, flexible structure Overlaying disulfides (C_600–635_; C_630–640_): abrogates membrane binding and substrate recognition	PDI catalyzes the isomerisation from the linear to the overlaying disulfide pattern.	[135, 141]

Ask1	C_200, 250_: intramolecular disulfide C_250_: interaction with Trx1	ASK1 is involved in TLR4 signaling and is involved in TNF*α*-induced apoptosis. Intramolecular disulfide induces conformational changes within the Trx-binding region.	Trx1 and Grx1 bind to ASK1 and inhibit the kinase; in case of Trx1 proteasomal degradation is induced. Oxidation of Trx1/ Grx1 induces the dissociation of the complex and kinase activation.	[14, 96, 97, 257]

EGFR	C_797_: sulfenylation	EGFR-mediated signaling; sulfenylation enhances tyrosine kinase activity.	Oxidation by H_2_O_2_	[90, 91]

HMGB1	C_23_, C_45_, C_106_: intramolecular disulfide (C_23–45_), sulfenylation(C_106_)	Fully reduced: chemotactic activity; intramolecular disulfide (C_23–45_), reduced C_106_: cytokine	Trx1 (Grx1?)	[154, 155, 157]

Myd88	8 Cys residues: (i) intermolecular disulfides (ii) nitrosylation	Intermolecular disulfides: oligomerisation during TLR signaling	Oxidation by H_2_O_2_ (Prx?), Nrx, Trx	[21, 93, 94]

NF*κ*B	C_62_: (i) glutathionylation (ii) sulfenylation	Reduced C_62_: DNA binding and gene expression	Bound in an inactive complex by Trx1 (cytosol), reduction by Trx1, Grx1 (nucleus)	[16, 99, 101]

Src	C_245_, C_487_: disulfide formation	Intramolecular disulfide connects SH2 and kinase domain and stabilizes the active conformation of the kinase	Oxidation by H_2_O_2_	[88, 89]

**Table 2:** Clinical implications of redox enzymes.

Protein	Reactive species	Pathology	Levels/role	Reference
Myeloperoxidase	Production of hypochlorous and hypobromous acid	Alzheimer's disease, Parkinson's disease	Beneficial	[172, 258]
		Arteriosclerotic plaques	Increased	[259]
		Breast cancer and chemotherapy	Activity increased/beneficial	[177]
		Cardiovascular disease	Increased (plasma)	[165]
		Chronic lung infection in preterm infants	Increased (tracheal aspirates)	[170]
		Rheumatoid arthritis	Increased (plasma, synovial fluid)	[168]
Lactoperoxidase	Production of hypothiocyanate	Chronic peridontitis	Oral LPO administration had no effect on disease	[179]
		Peridontitis in diabetes mellitus type I	Activity increased (saliva)	[180]
		Recurrent aphtous stomatitis	Decreased (saliva)	[183]
		Smoking	Activity decreased (saliva)	[187]
NADPH oxidase	Production of superoxide and secondary hydrogen peroxide	Acute myocardial infarct	Increased (heart tissue), activity increased (heart tissue), increased (saphenous vein)	[192–194]
		Cardiovascular disease	Increased (serum)/detrimental	[202]
		Chronic granulomatous disease	Activity decreased (peripheral blood neutrophils)/detrimental	[189]
		Diabetes nephropathy	Increased/phase II trial completed	[199]
		Melanoma	Similar (melanoma tissue)/no correlation with invasiveness	[201]
		Retinopathy	Increased/detrimental	[260]
Nitric oxide synthase	Production of nitric oxide	Asthma	Inhibition detrimental/inhibition beneficial/inhibition had no effect	[224–226]
		Breast cancer	Increased/none	[234]
		Head and neck cancer	Increased/detrimental (in respective cancer tissue)	[235]
		Heart disease and rejected transplants	Increased (heart tissue)	[228–232]
		Melanoma	Increased/detrimental	[233]
		Migraine	Inhibition beneficial/inhibition had no effect	[220–222]
		Rheumatoid arthritis	Increased/inhibition beneficial	[227]
		Sepsis	Inhibition detrimental/beneficial/no effect (serum)	[213–217]
Peroxiredoxins	Decomposition of H_2_O_2_, redox signaling	Alzheimer's disease	Prx1/Prx2 increased (brain tissue), Prx2 activity decreased (blood), Prx3 decreased (brain tissue)	[241–243, 248]
		Cataracts	Prx6 decreased (eye tissue)	[250]
		Diabetes mellitus type II	Prx4 increased (serum)	[252]
		Diabetic retinopathy	Prx1 increased (vitreous biopsy)	[251]
		Glaucoma	Prx6 increased (eye tissue)	[249]
		Lung cancer	Prx1 increased, Prx3 increased (cancer tissue)	[254, 255]
		Parkinson's disease	Prx2 increased	[244]

## Abbreviations

ADAM17: A disintegrin and metalloproteinase 17
DAMP: Damage-associated molecular pattern
DUOX: Dual oxidase
EGF: Epidermal growth factor
FAD: Flavin-adenine dinucleotide
Gpx: Glutathione peroxidase
Grx: Glutaredoxin
HMGB1: High-mobility group protein 1
IL: Interleukin
IRAK: Interleukin-1 receptor-associated kinase
LPO: Lactoperoxidase
LPS: Lipopolysaccharide
MAPK: Mitogen-activated protein kinase
MIF: Macrophage migration inhibitor factor
MPD: Membrane-proximal domain
MPO: Neutrophil-derived myeloperoxidase
Myd88: Myeloid differentiation primary response 88
NLR: NOD-like receptors
NOS: Nitric oxide synthase
NOX: NADPH oxidase
Nrx: Nucleoredoxin
PAMP: Pathogen-associated molecular pattern
Prx: Peroxiredoxin
SOD: Superoxide dismutase
TIR: Toll/IL-1 receptor
TNF: Tumor necrosis factor
TLR: Toll-like receptor
Trx: Thioredoxin
Txnip: Thioredoxin-interacting protein
ROS: Reactive oxygen species
RNS: Reactive nitrogen species.
